# Large language models in sports injury care: a comparative expert evaluation of GPT-4o and GPT-5

**DOI:** 10.1186/s13102-026-01675-z

**Published:** 2026-03-30

**Authors:** Onur Kaya, Gazi Huri, Emre Anıl Özbek, Nevzat Gönder, İbrahim Halil Demir, Kaan Ali Dalkır

**Affiliations:** 1 Department of Orthopaedics and Traumatology, Gaziantep City Hospital, Gaziantep, Türkiye; 2 Department of Orthopaedics and Traumatology, Aspetar, FIFA Medical Center of Excellence, Doha, Qatar; 3 Department of Orthopaedics and Traumatology, Ankara University, Ankara, Türkiye; 4 Department of Orthopaedics and Traumatology, Gaziantep University, Gaziantep, Türkiye; 5 Department of Orthopaedics and Traumatology, Cukurova University, Adana, Türkiye

**Keywords:** large language models, GPT-4o, GPT-5, Sports injuries, Orthopedic decision-making, Artificial intelligence

## Abstract

**Background:**

Large language models (LLMs) have shown increasing relevance in clinically supervised decision-support frameworks; however, their performance in orthopedic sports injury scenarios remains unclear. This study aimed to comparatively evaluate the diagnostic, treatment, and rehabilitation recommendations generated by GPT-4o and GPT-5 using standardized clinical scenarios assessed by orthopedic specialists.

**Methods:**

Fifteen sports injury–based clinical scenarios were developed and validated by orthopedic specialists with subspecialty expertise in sports traumatology. Each scenario was scored for clinical realism, adequacy of physical examination findings, and adequacy of radiological information using a 7-point Likert scale adapted from AGREE II domains. Both GPT-4o and GPT-5 were prompted using standardized zero-shot instructions, with each scenario submitted three times to assess internal consistency. Two blinded orthopedic specialists evaluated content-level consistency, and five independent orthopedic specialists scored the expert-rated clinical adequacy of AI-generated responses on a 0–5 scale. Inter-rater reliability was assessed using the intraclass correlation coefficient (ICC) and Cohen’s kappa.

**Results:**

Specialists rated the clinical scenarios favorably, with 69–72% agreement across domains and ICC values indicating good reliability for clinical realism (ICC = 0.725) and moderate reliability for physical examination (ICC = 0.634) and radiological adequacy (ICC = 0.512). GPT-4o produced consistent outputs in 93.3% of cases, with one scenario showing clinically relevant inconsistency (κ = 0.82). Comparative expert evaluation demonstrated significantly higher scores for GPT-5 (median = 4.60) than GPT-4o (median = 4.00) (*p* = 0.007). Inter-rater reliability for AI response scoring was high for both models (ICC = 0.888 for GPT-4o; ICC = 0.895 for GPT-5).

**Conclusion:**

GPT-4o and GPT-5 generated responses with generally high expert-rated clinical adequacy and strong consistency in standardized sports injury–related clinical scenarios, with GPT-5 achieving higher scores in expert evaluations. By providing a structured, specialty-specific expert assessment under controlled conditions, this study adds comparative insight into how contemporary large language models are perceived in orthopedic sports injury contexts, without implying objective diagnostic accuracy or autonomous clinical decision-making.

**Supplementary Information:**

The online version contains supplementary material available at 10.1186/s13102-026-01675-z.

## Introduction

With the advancement of technology, artificial intelligence (AI) has made significant progress in many fields. AI, which may mark the beginning of a new era that we have yet to fully comprehend, has begun to be utilized and tested in the healthcare sector. In medicine, artificial intelligence is increasingly being used in various processes such as diagnostic support, radiology, surgical planning, and patient education. One of the most commonly used AI tools, ChatGPT (Generative Pre-trained Transformer) (OpenAI, San Francisco, CA), is a generative language model developed by OpenAI. It has the capacity to engage in conversation through a text-based interface with an extensive AI network. Within just two months of its release, ChatGPT reached approximately 100 million users and rapidly became one of the fastest-growing internet-based technologies, attracting around 1.5 billion visitors per month [1, 2].

The use of artificial intelligence in healthcare has become increasingly common, not only for answering patients’ questions but also for assisting healthcare professionals in optimizing clinical management [3]. In recent years, an approach known as prompt engineering has played a critical role in improving the quality and reliability of outputs obtained from large language models (LLMs). The quality of the output generated by LLMs largely depends on the design of the prompts; therefore, developing clear, contextual, and purpose-oriented prompts is crucial to enhancing the reliability and quality of AI outputs in the healthcare domain [4].

In this context, prompt engineering approaches have also begun to be integrated into the generation of case scenarios that are widely used in clinical education. The process of scenario development generally involves synthesizing data obtained from real patient cases, current clinical guidelines, and expert consensus. Well-structured scenarios provide a controlled framework that allows systematic evaluation of responses to clinically meaningful and contextually rich information, thereby enabling the assessment of decision-making performance [5, 6].

Large language models (LLMs), which offer patients an innovative communication tool during the treatment decision-making process, hold the potential to facilitate clinical workflows and support treatment planning in specialties such as orthopedics. However, their clinical integration remains limited due to challenges related to domain specificity, validation, and reliability. They demonstrate promising potential when the decision-making process involves the integration of imaging data, procedural guidelines, and patient-specific variables [7].

Although performance improvements across successive generations of large language models are generally expected, whether such improvements translate into expert-perceived clinical adequacy remains unclear, particularly in specialty-specific domains such as sports medicine. Comparative evaluations between model generations are therefore meaningful to explore how incremental model advances are reflected in expert assessments within standardized clinical scenarios.

In addition, although advanced approaches such as Retrieval-Augmented Generation (RAG) and domain-specific fine-tuning may enhance model performance, the present study intentionally focused on evaluating the baseline performance of general-purpose LLMs under standardized conditions.

The aim of this study was to evaluate the expert-rated clinical adequacy of diagnostic, treatment, and rehabilitation recommendations generated by large language models in response to predefined sports medicine clinical scenarios.

## Materials and methods

This study was conducted with a cross-sectional analytical design to analyze the expert-rated adequacy of artificial intelligence–generated diagnostic and treatment responses in sports injury–related clinical scenarios, based on evaluations by orthopedic specialists.

### Scenario development and expert validation

Clinical scenarios were designed based on a comprehensive review of the current literature and clinical data obtained from real patient cases. The scenarios were selected to represent a range of common sports injury presentations encountered in orthopedic practice, rather than to achieve a balanced distribution across anatomical regions or specific pathologies. The drafts were initially evaluated by two orthopedic specialists with a subspecialty focus in sports traumatology, each with at least five years of professional experience, to ensure content integrity and clinical applicability. The number of scenarios was determined to provide sufficient clinical variability while maintaining feasibility for repeated AI querying and expert evaluation. There are no conflicts of interest between the authors and the evaluating specialists in this study.

After necessary revisions, consensus was achieved on fifteen finalized scenarios. Each scenario included patient history (age, sex, type of sport, mechanism of injury), physical examination findings (special tests, range of motion), and radiological results (X-ray, MRI, CT).

The scenarios were evaluated by five orthopedic specialists, each with at least five years of experience, with a subspecialty focus in sports traumatology, under three main categories: Clinical Realism, Adequacy of Physical Examination Findings, and Adequacy of Radiological Information. These categories were adapted from the Scope and Purpose, Clarity of Presentation, and Applicability domains of the AGREE II instrument. Each domain was scored using a 7-point Likert scale (1 = strongly disagree, 7 = strongly agree) [8].

The inter-rater reliability among the five orthopedic specialists who rated the clinical scenarios was analyzed using the Intraclass Correlation Coefficient (ICC [2,1]), calculated with a two-way random-effects, absolute agreement model. The distribution of scores assigned by five independent specialists for each scenario in the domains of clinical realism, adequacy of physical examination findings, and adequacy of radiological information was visualized through a heatmap analysis .

This study was designed as an exploratory analysis, consistent with similar LLM evaluation studies in the literature. Having five orthopedic specialists independently rate the same fifteen scenarios increased per-unit information yield within a paired design framework. Since the primary objective was to detect clinically meaningful rather than small statistical differences, no a priori power analysis was performed; instead, 95% confidence intervals were reported to present uncertainty related to effect size.

### Retrieval of AI responses and consistency analysis

In the second phase, the same fifteen scenarios were presented to the GPT-4o and GPT-5 models. Each clinical scenario was introduced to the models using carefully calibrated and structured zero-shot prompts without providing any example responses (Table 1). To evaluate the consistency of the outputs, each prompt was submitted to each model three times under identical conditions, and the internal consistency of the responses was examined.

**Table 1:** Standardized prompt structure for clinical scenarios

Component	Content
Case [No]	Sequential case number
Age	[X years]
Gender	Male / Female
Sport	Type of sport and competition level
History	Presenting complaint and relevant medical/sports history
Physical Examination	Findings from clinical examination (e.g., ROM, special tests, tenderness)
Radiology	Relevant imaging findings (X-ray, MRI, CT, etc.)
Instruction to AI model	“Based on the clinical scenario above, please provide: 1. The most likely diagnosis. 2. The recommended treatment plan (conservative and/or surgical options if applicable). 3. The estimated return-to-sport timeline.”

The internal consistency of the responses was reviewed by two independent orthopedic specialists, each with at least five years of experience, at the content level. The consistency assessment aimed to measure the reproducibility of the models when generating multiple responses to the same clinical scenario. During the consistency analysis phase, two orthopedic specialists independently evaluated the AI-generated responses in a blinded manner, without knowing which model (GPT-4o or GPT-5) produced each output. All responses were presented in an anonymous and randomized format to minimize potential evaluator bias.

Minor variations in wording or phrasing were considered insignificant. A response was marked as “inconsistent” when clinically meaningful differences were identified in the diagnosis, treatment approach, or rehabilitation recommendations. A discrepancy was considered clinically meaningful when differences affected the primary diagnosis, the overall treatment strategy (e.g., conservative versus surgical management), or return-to-sport recommendations. Minor variations in wording or equivalent treatment options were not considered inconsistencies.

Consistency evaluation was performed using a binary classification system (yes = consistent / no = inconsistent), and the Consistency Rate (%) was calculated using the formula: Consistency Rate (%) = (Number of Consistent Responses / Total Number of Scenarios) × 100.

The evaluations were independently conducted by two orthopedic specialists, and inter-observer agreement was analyzed using the standard (unweighted) Cohen’s kappa coefficient. Because the consistency assessment was based on a binary classification system (consistent vs. inconsistent), the use of the unweighted kappa statistic was considered appropriate. Since, in clinical applications, the initial response is typically the one considered by practitioners, only the first response generated by each model was included in the subsequent expert evaluation.

### Expert evaluation of AI responses

In the third phase, the responses generated by GPT-4o and GPT-5 were sent to five different orthopedic specialists each with at least five years of experience who also had experience in sports-related injuries. The evaluators were informed that the responses were generated by artificial intelligence; however, to minimize bias, the responses were presented in a standardized format without including any sources or technical details. Each model output was evaluated independently rather than through side-by-side comparison. For a given clinical scenario, GPT-4o and GPT-5 responses were presented in separate evaluation forms, and the specialists were blinded to the alternative model’s output at the time of assessment. The specialists were instructed to use their clinical experience during the categorization process but were encouraged to consider current clinical guidelines and evidence-based treatment approaches whenever possible. Additionally, the latest ESSKA recommendations and relevant literature summaries were provided to the specialists as reference material during the evaluation process.

The evaluation was conducted using a standardized evaluation table that allowed scoring on a 0–5 scale, where 0 = Completely non-compliant and 5 = Full compliance, with five main rating categories in between. The scores obtained were compared on a case-by-case basis, and performance differences between the two models were analyzed using statistical methods.

GPT-4o and GPT-5 models were accessed exclusively via the ChatGPT web interface (OpenAI, San Francisco, CA) rather than through the Application Programming Interface (API). No fine-tuning, Retrieval-Augmented Generation (RAG), or external data augmentation was applied. All model interactions were conducted under standardized conditions using a fixed system message and case-specific user prompts. The models were accessed through the ChatGPT web interface under default system-controlled inference settings. Each clinical scenario was submitted in independent sessions to avoid contextual carryover effects. Detailed prompt structures, inference parameters, model selection procedure, and example model outputs are provided in Supplementary File 1 to ensure transparency and reproducibility, including the manual selection of GPT-4o and GPT-5 via the ChatGPT web interface at the time of data collection.

Supplementary File 1 includes the standardized prompts used in the study, the first responses of GPT-4o and GPT-5 for each clinical scenario, identical ESSKA consensus guideline materials (e.g., the 2016 ESSKA Consensus Conference recommendations on meniscal lesions) and related literature summaries provided to the specialists as an evidence-based reference framework, and a detailed explanation of the 0–5 scoring scale used in the evaluation process.

In the present study, no single objective ‘ground truth’ was defined in terms of patient outcomes or diagnostic accuracy. Instead, AI-generated responses were evaluated against expert judgment, which was intentionally used as the primary reference standard. To anchor expert assessments to an evidence-based framework and reduce subjectivity, evaluators were provided with the most recent ESSKA recommendations and relevant literature summaries, and clinical scenarios were constructed to reflect guideline-consistent presentations. Expert assessments were performed independently and in a blinded manner by multiple specialists, and high inter-rater reliability was observed.

### Statistical analysis

The agreement among the five specialists regarding the ratings of clinical realism, adequacy of physical examination findings, and adequacy of radiological information in the scenarios was evaluated using the Percent Agreement method to indicate the percentage of exact consensus among raters. The internal consistency of the responses was analyzed using Cohen’s kappa coefficient to measure the level of agreement between the two independent evaluators. The reliability of the ratings provided by the five specialists for each case was examined using the Intraclass Correlation Coefficient (ICC [2,1], two-way random effects, absolute agreement) model. For comparison of the case-based scores between the GPT-4o and GPT-5 models, the non-parametric Wilcoxon signed-rank test was used. Because the ratings were based on an ordinal Likert-type scale (0–5), a non-parametric approach was considered more appropriate.

The significance level was 0.05, with a confidence interval of 95%. Statistical analyses were performed using the SPSS for Windows, version 22.0 software package.

## Results

**Fig. 1:**
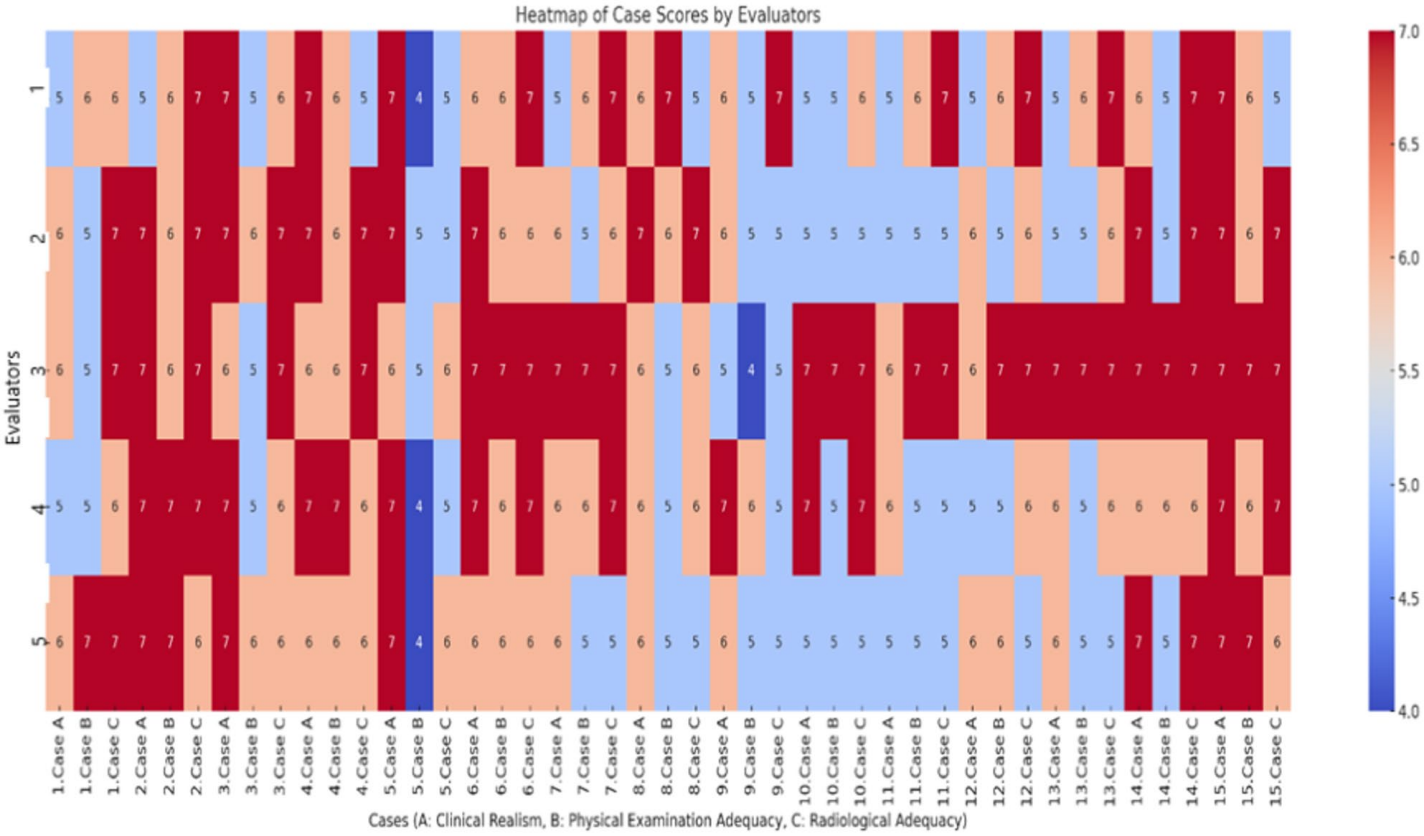
Heatmap showing specialists’ ratings (range: 4–7) across 15 clinical scenarios (**A** Clinical Realism, **B** Physical Examination Adequacy, **C** Radiological Adequacy). Each column represents one scenario, and each row corresponds to one of the five independent orthopedic specialists (indexed 1–5). The color scale reflects the assigned scores, with darker red tones indicating higher ratings and blue tones indicating lower ratings

The evaluations of five independent orthopedic specialists regarding the adequacy of the scenarios in terms of clinical realism, physical examination findings, and radiological information revealed that 1.8% of the ratings were 4, 28.9% were 5, 32.9% were 6, and 36.4% were 7 (Fig. 1).

Inter-rater agreement among the specialists was analyzed using the Percent Agreement method and Intraclass Correlation Coefficient (ICC [2,1]) method. The mean agreement rate was 72.2% for clinical realism, 68.5% for the adequacy of physical examination findings, and 70.1% for the adequacy of radiological evaluation. The ICC values were 0.725 for clinical realism, 0.634 for the adequacy of physical examination findings, and 0.512 for the adequacy of radiological information; all values were found to be statistically significant (*p* < 0.05).(Table 2) These results indicate that the clinical scenarios demonstrated an overall satisfactory level of inter-rater reliability. The reliability was good for Clinical Realism, and moderate for both Physical Examination and Radiological Adequacy. Overall, the findings suggest that the scenarios were clinically well-constructed and consistently interpreted by the participating specialists.

**Table 2:** Inter-rater reliability of specialists’ evaluations for clinical scenarios (Intraclass Correlation Coefficients)

	ICC	95% Confidence Interval		
		Lower Bound	Upper Bound	*P*
Clinical Realism	0.725	0.421	0.895	0.001*
Physical Examination	0.634	0.227	0.860	0.004*
Radiological Adequacy	0.512	0.030	0.814	0.030*

*p < 0.05

For each clinical scenario, three separate outputs were generated from the same model and compared in terms of diagnosis, treatment approach, and rehabilitation plan.

Among the fifteen scenarios, fourteen demonstrated mutually consistent responses, with only minor wording differences that were considered clinically insignificant. However, in Case 11, one of the three GPT-4 outputs showed a discrepancy in the treatment recommendation, which was evaluated as inconsistent. It was found that in 14 out of 15 cases (93.3%), the responses were 100% consistent, while in one case, 80% similarity was observed. Inter-observer agreement was analyzed using Cohen’s kappa coefficient, which was calculated as κ = 0.82.

**Fig. 2:**
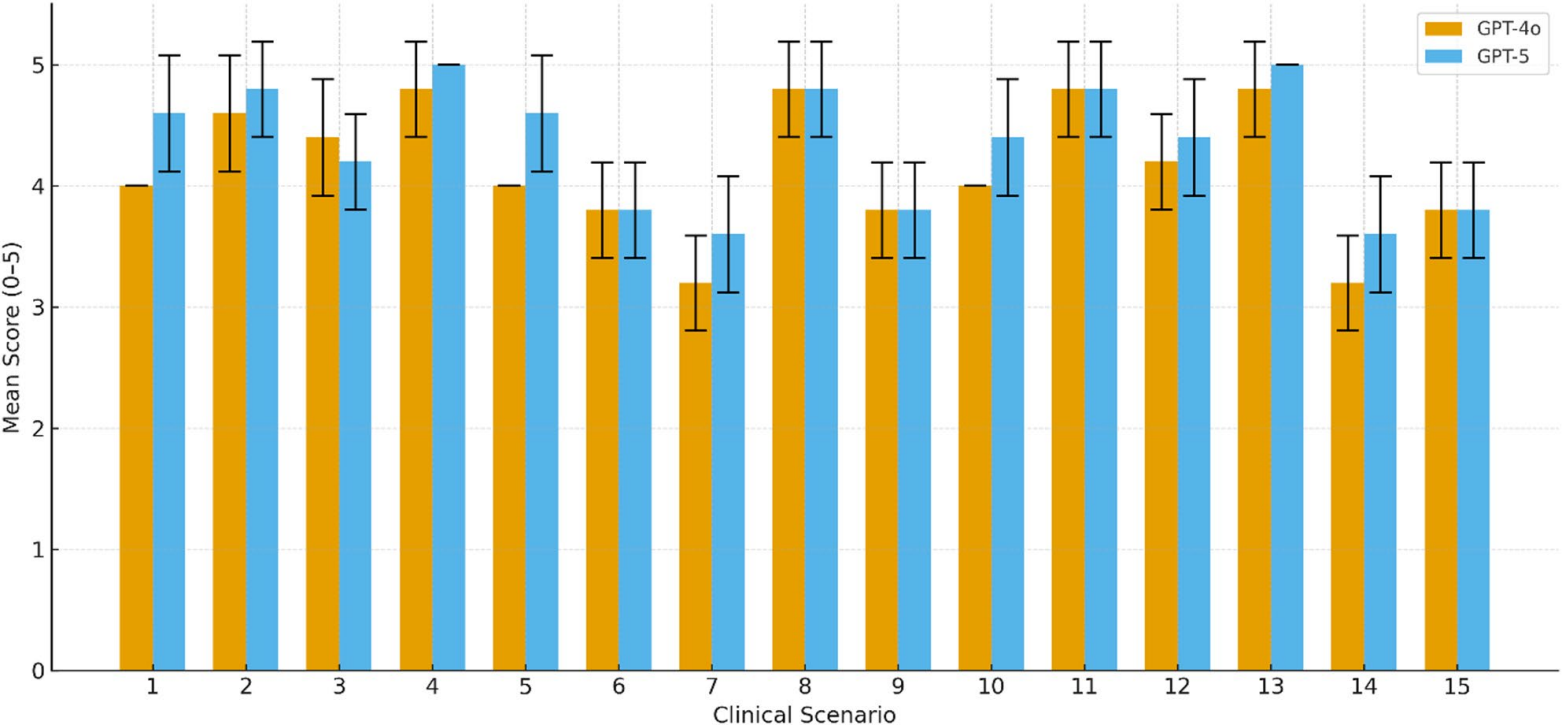
Per-case mean scores for GPT-4o and GPT-5 with 95% confidence intervals. Each bar represents the mean rating assigned by five independent orthopedic specialists for each clinical scenario, and the capped error bars indicate the 95% confidence intervals based on the distribution of individual ratings

A total of 15 clinical scenarios developed in the study were independently evaluated by five orthopedic specialists. For each case, the responses generated by GPT-4o and GPT-5 were separately rated (Fig. 2).

A statistically significant difference was found between the overall performances of GPT-4o and GPT-5. According to the Wilcoxon signed-rank test, the median score for GPT-4o was 4.00 (IQR: 3.80–4.80), while GPT-5 achieved a higher median score of 4.60 (IQR: 3.80–4.80) (*p* = 0.007). The Hodges–Lehmann median paired difference between GPT-5 and GPT-4 was 0.20 points (95% CI: 0.10–0.30), corresponding to a large effect size (rank-biserial correlation *r* = 0.89). In addition to p-values, effect sizes were calculated using rank-biserial correlation, and median paired differences with 95% confidence intervals were estimated using the Hodges–Lehmann approach.(Table 3).

**Table 3:** Comparative summary of expert ratings for GPT-4o and GPT-5 Across 15 clinical scenarios

	Median	25th percentile	75th percentile	P	Median paired difference (95% CI)	Effect size (r)
GPT4	4.00	3.80	4.80	0.007*	-	-
GPT5	4.60	3.80	4.80		0.20 (0.10–0.30)	0.89

*p < 0.05, Wilcoxon signed-rank test

This finding indicates that GPT-5 was rated higher by orthopedic specialists in terms of response quality and clinical adequacy. The inter-rater reliability among the five orthopedic specialists who scored the AI-generated responses was evaluated using the Intraclass Correlation Coefficient (ICC [2,1]), based on a two-way random-effects, absolute-agreement model. The ICC values were 0.888 (95% CI: 0.763–0.957) for GPT-4o and 0.895 (95% CI: 0.779–0.960) for GPT-5, and both were statistically significant (*p* < 0.05). (Table 4) These results demonstrate a high level of consistency among evaluators for both models and indicate that GPT-5 achieved slightly higher mean scores while maintaining comparable inter-rater reliability, suggesting more stable and clinically reliable responses.

**Table 4:** Comparison of inter-rater reliability between GPT-4o and GPT-5 evaluations (ICC [2,1] analysis)

	ICC	95% Confidence Interval			
		Lower Bound		Upper Bound	*P*
GPT-4	0.888	0.763		0.957	0.001*
GPT-5	0.895		0.779	0.960	0.001*

*p < 0.05

## Discussion

The integration of artificial intelligence into clinical decision-support processes is becoming increasingly important. This study aims to fill a significant gap in the literature by directly comparing the clinical adequacy of the GPT-4o and GPT-5 models in scenarios specific to sports injuries.

OpenAI models benefit from reinforcement learning with human feedback, enabling them to generate responses that are more consistent with clinical guidelines—a feature that enhances their potential in clinical practice [9]. In our study, the evaluations based on expert feedback were also grounded in current clinical guidelines.

In the literature, it has been emphasized that feedback from senior clinicians is a critical step for the correct and acceptable application of LLMs, and that clinicians should receive training on effective prompt writing and critical evaluation of AI-generated outputs [10]. Similarly, in our study, the prompts used to generate clinical scenarios were evaluated by experienced orthopedic specialists, highlighting the importance of expert validation.

In a study on total knee arthroplasty (TKA), LLM responses were rated using a five-point Likert scale to assess the accuracy of patient-communication responses, based on evaluations by four orthopedic surgeons [11]. In our study, a seven-point Likert scale and evaluations by five specialists were used. Therefore, both studies relied on expert opinion and comparable scoring approaches.

Among the risks associated with emerging technologies are AI hallucinations, fabrication of information, and overconfidence. Such errors often arise not from structural deficiencies of LLMs themselves but from improper use, which underscores the growing importance of well-designed prompt construction [12].

It is noteworthy that studies simulating clinical scenarios in the field of orthopedics to test AI-generated responses remain very limited in the current literature. In other medical disciplines, for instance, one study examined ChatGPT’s capacity to evaluate the relationship between concussion, repetitive brain trauma, and the risk of neurodegenerative diseases. This study highlighted several deficiencies of ChatGPT, including issues related to accuracy, transparency, bias, manipulation, integrity, and response delay [13].

Similarly, a recent study evaluated the diagnostic accuracy of various LLMs and demonstrated that prompting strategies (e.g., zero-shot, few-shot) play a decisive role in model performance [14]. In our study, however, orthopedic sports injury–specific clinical scenarios were presented in a standardized format without providing sample answers, thereby adopting a zero-shot prompting approach. This method ensured that the responses were not guided and allowed for a more objective assessment of consistency.

In another study investigating the diagnostic accuracy of AI in patients with knee pain, high accuracy was reported; however, it was also emphasized that clinically significant errors might occur depending on the provided information and guidance strategy [15]. In our study, to minimize such an influence, each scenario was presented to both models three times using the same prompt, and the consistency of the responses was analyzed.

A study covering various orthopedic subspecialties reported that models demonstrated higher performance in basic sciences, attributed to the availability of standardized and well-structured reference materials. In contrast, trauma and pediatrics showed substantial gaps due to variable clinical presentations, complex decision-making processes, and the lack of standardized guidelines [16, 17]. Conversely, in our study, which focused on another orthopedic subspecialty—sports injuries—we found that AI provided highly successful responses when evaluated according to the most recent ESSKA recommendations and relevant literature.

One study demonstrated that ChatGPT-4 was able to accurately diagnose foot and ankle cases, provide satisfactory treatment recommendations, and maintain consistency among surgeon evaluators. Moreover, unlike earlier studies that evaluated ChatGPT-3.5, it was emphasized that ChatGPT-4 did not suggest non-existent treatment options or present fabricated information as factual [18]. In our study as well, the responses generated by GPT-4o and GPT-5 were found to be generally consistent according to expert evaluations, and the provision of literature references to the specialists helped maintain objectivity. This finding suggests that updates in AI models contribute to improvements in both consistency and accuracy.

Another study utilized AI-generated case scenarios designed to produce responses aligned with a country’s existing healthcare policies and based on competencies such as attitude, communication, and ethics. The study concluded that, given its acceptable level of accuracy, artificial intelligence could potentially be used in medical education, particularly in countries facing a shortage of qualified instructors [19]. Our study supports this view, indicating that while the models provided accurate and educationally valuable responses in the evaluation of clinical scenarios, it should be recognized that they may evolve over time and might not always align with the most current clinical guidelines.

A similar study selected ten common orthopedic trauma cases from OrthoBullets, assigning GPT-4 the task of interpreting medical imaging and patient data to generate diagnoses. The AI-generated responses were subsequently evaluated by four orthopedic surgeons. Although the quality of the AI’s responses was found to be below the expected clinical standard, the authors suggested that, with continued advancements, artificial intelligence could eventually be integrated into medical education [20].

In another study, ChatGPT was used to create personalized rehabilitation plans for elderly patients, highlighting several advantages such as easy accessibility, individualized conversational interactions, and reduced need for additional staff [21]. In our study, both GPT-4o and GPT-5 systematically suggested widely accepted rehabilitation protocols for sports injuries. However, because the models did not account for athletes’ individual characteristics, their responses demonstrated a limited capacity for personalization.

Finally, a study emphasized that artificial intelligence tools cannot replace physicians due to limitations related to medical ethics, accountability, legal responsibilities, data interpretation, and anatomical variability [22]. At the same time, recent literature has highlighted that structured prompting strategies—such as zero-shot, few-shot, and chain-of-thought prompting—can substantially influence the reliability, interpretability, and clinical applicability of large language models in healthcare settings [23, 24]. Although GPT-4o and GPT-5 produced responses consistent with general clinical protocols, their limitations in areas such as individualized return-to-sport planning, complication management, and patient-specific treatment adjustment indicate that they should be regarded solely as complementary decision-support tools under expert supervision in the context of sports injuries. Although GPT-5 demonstrated higher median scores than GPT-4o, this difference reflects expert-perceived clinical adequacy under standardized conditions and should not be interpreted as evidence of a clinically meaningful impact on patient management or decision-making. Inappropriate application of such outputs without expert oversight may result in clinically relevant consequences, including delayed diagnosis, inappropriate management decisions, or premature return to sport and reinjury.

### Limitations

This study has several limitations. First, the clinical scenarios were artificially constructed and may not fully capture the complexity, missing information, comorbidities, and decision uncertainty encountered in real-world clinical practice. Second, as no standardized objective gold reference was available to evaluate the accuracy of AI-generated responses, the analyses relied primarily on expert judgment, which may introduce subjective bias despite the use of multiple blinded evaluators and high inter-rater reliability. Third, the limited number of participating specialists may reduce the statistical power and generalizability of the findings.

In addition, a structured error taxonomy and harm grading system was not applied, limiting the ability to quantify the clinical severity of specific model errors. Furthermore, as the evaluated models (GPT-4o and GPT-5) are continuously updated systems, the findings reflect model performance during a specific data collection period and may vary over time. Finally, this study assessed expert-perceived clinical adequacy rather than objective diagnostic accuracy or patient outcomes, which limits direct inference regarding the true diagnostic or therapeutic performance of the models.

Accordingly, the potential utility of LLMs should be confined to supervised, clinician-facing contexts such as education, training, and information support, rather than autonomous clinical decision-making.

Another limitation of this study is that data were collected during a period when explicit model selection was available in the ChatGPT web interface. As the platform continues to evolve, such features may not remain consistently accessible, which may limit direct reproducibility under identical interface conditions. However, this also reflects the dynamic nature of large language model development and highlights the importance of time-specific evaluations when assessing model performance.

## Conclusion

This study demonstrates that GPT-4o and GPT-5 generate responses that are generally rated as clinically adequate and consistent by orthopedic specialists when applied to standardized sports injury–related clinical scenarios. By focusing on expert-based evaluation under controlled conditions, the findings provide a specialty-specific perspective on how contemporary large language models perform in structured clinical contexts. Although these results do not imply objective diagnostic accuracy or direct clinical decision-making capability, they offer valuable insight into the perceived reliability and limitations of LLM-generated recommendations, thereby contributing to the ongoing discussion on their appropriate and supervised use in clinical practice.

## Supplementary Information

Supplementary Material 1.

## Data Availability

All data generated or analyzed during this study are included in this published article and its supplementary files.
