# Supplementary material for: Large language models in sports injury care: a comparative expert evaluation of GPT-4o and GPT-5
Source: BMC Sports Sci Med Rehabil. 2026 Mar 30;18:232. doi: 10.1186/s13102-026-01675-z (PMC13154586; doi:10.1186/s13102-026-01675-z)
Supplement: Supplementary file 1 — Supplementary Material 1. [file 13102_2026_1675_MOESM1_ESM.docx]

**Supplementary File – Standardized Prompts Used for GPT-4o and GPT-5 Evaluation**

**Introduction**To ensure transparency and reproducibility, the exact prompts used in this study for generating GPT-4o and GPT-5 responses are provided in full.
Each clinical scenario was entered into the AI model using a standardized system message and a case-specific user message.
This approach ensured that all cases were presented under the same instructional conditions, minimizing variability introduced by prompt phrasing.

**Method of Prompt Use**

Model Selection Procedure:

At the time of data collection, model selection was performed manually via the ChatGPT web interface using the model selection menu located in the upper-left panel. This allowed explicit selection between available model versions (GPT-4o and GPT-5). Each clinical scenario was entered into separate chat sessions after confirming the active model.

Platform: ChatGPT web (OpenAI, San Francisco, CA)

Models:

• GPT-4o (OpenAI, San Francisco, CA; model snapshot: not publicly versioned)

• GPT-5 (OpenAI, San Francisco, CA; model snapshot: not publicly versioned)

Date of Access (query window): 10 August 2025 (Europe/Istanbul, UTC+3)

System Message:

Identical for all cases (see below)

User Message:

Contained case-specific patient demographics, history, physical examination findings, and radiology reports (without explicit final diagnosis).

Session Isolation:

Each case was submitted in an independent chat session.

Repetition Protocol:

• Each case was submitted three separate times in independent sessions per model.

• For subsequent analyses, the first response from each model was used for evaluation.

Evaluator Blinding:

Orthopedic sports medicine specialists were informed that responses were AI-generated. Responses were presented in a standardized format without sources or technical metadata.

Inference Parameters:

Inference parameters such as temperature and maximum token limits are internally controlled by the ChatGPT web interface and cannot be explicitly modified by the user. Therefore, no manual parameter configuration was applied during the generation of model responses.

**Standardized Prompt Structure for Clinical Scenarios**
Case [No]
Age: [X years]
Gender: [Male/Female]
Sport: [Type of sport, competition level]
History: [Presenting complaint and relevant history]
Physical Examination: [Findings from clinical examination]
Radiology: [Relevant imaging findings]

**Instruction to AI model:**
“Based on the clinical scenario above, please provide:

1. The most likely diagnosis.
2. The recommended treatment plan (conservative and/or surgical options if applicable).
3. The estimated return-to-sport timeline.”

# GPT-4o and GPT-5 Evaluation Table

| Model | Score | Definition |
| --- | --- | --- |
| GPT-4o | 0 | Completely non-compliant |
|  | 1 | Very low compliance |
|  | 2 | Low compliance |
|  | 3 | Moderate compliance |
|  | 4 | Good compliance |
|  | 5 | Full compliance |
| GPT-5 | 0 | Completely non-compliant |
|  | 1 | Very low compliance |
|  | 2 | Low compliance |
|  | 3 | Moderate compliance |
|  | 4 | Good compliance |
|  | 5 | Full compliance |

**Case 1**

**Age:** 32 years
**Gender:** Male
**Sport:** Professional football player (right-foot dominant)
**History:** Intermittent right knee pain worsening over the past week.
**Physical Examination:** Medial McMurray test (+), normal range of motion but pain in flexion.
**Radiology:** X-ray shows 15° varus deformity bilaterally. MRI shows posterior root tear of the medial meniscus (right knee).

**ESSKA recommendation**
“In patients with meniscal root tears associated with varus malalignment, corrective osteotomy should be considered to improve the mechanical environment for meniscal healing and prevent further joint degeneration.”

**References**
Beaufils P, Pujol N, Michaut M, et al. Clinical practice guidelines for the management of meniscus lesions and disorders: The 2016 ESSKA Consensus Conference. *Knee Surg Sports Traumatol Arthrosc.* 2017;25(2):335–346. <https://doi.org/10.1007/s00167-016-4400-3>

**GPT-4:**

1. **Most Likely Diagnosis:** Posterior root tear of the medial meniscus in a varus-aligned knee.
2. **Recommended Treatment Plan:**
   - Surgical: Arthroscopic root repair ± high tibial osteotomy (HTO) due to varus alignment.
   - Conservative: Activity modification, physiotherapy, NSAIDs (less preferred in athletes).
3. **Estimated Return-to-Sport Timeline:**
   - Surgical: 4–6 months post-repair (if isolated); 6–9 months if combined with HTO.
   - Conservative: Varies, often not recommended due to progression risk.

**GPT-5:**

1. **Most likely diagnosis:** Posterior root tear of the medial meniscus (right knee) with bilateral varus alignment.
2. **Recommended treatment plan:**
   - Surgical: Arthroscopic medial meniscus root repair combined with high tibial osteotomy to correct varus deformity.
   - Conservative (if low demand): Activity modification, physiotherapy, unloader brace, NSAIDs.
3. **Estimated return-to-sport timeline:**
   - Phase 1 (0–6 weeks): Protected weight-bearing, ROM exercises.
   - Phase 2 (6–12 weeks): Progressive strengthening, proprioception training.
   - Phase 3 (3–6 months): Sport-specific drills, gradual running.
   - Full return: ~6–9 months post-surgery, depending on healing and functional recovery.

**Case 2**

**Age:** 41 years
**Gender:** Male
**Sport:** Professional grass-court tennis player
**History:** Right shoulder pain, intermittent for a month, worsening after the last match.
**Physical Examination:** Painful right shoulder movements, restriction in abduction, Hawkins test (+), Yergason test (−), Belly Press test (−), no scapular dyskinesia.
**Radiology:** X-ray normal. MRI shows partial tear in the bursal side of the supraspinatus tendon, edema around the biceps tendon groove, and degenerative changes in the acromioclavicular joint.

**Literature-Based Recommendation – Management of Partial-Thickness Rotator Cuff Tears**
“For partial-thickness rotator cuff tears, initial management should be non-operative, including activity modification, physiotherapy, and non-steroidal anti-inflammatory drugs (NSAIDs). Surgical intervention may be considered if symptoms persist beyond 3–6 months despite appropriate conservative measures.”
*Reference:* Abat F, et al. *J Exp Orthop.* 2017;4(1):18. doi:10.1186/s40634-017-0092-6

**GPT-4:**

1. **Most Likely Diagnosis:** Partial-thickness bursal-sided supraspinatus tear with AC joint degeneration and biceps groove irritation.
2. **Recommended Treatment Plan:** Initial conservative management—activity modification, NSAIDs, subacromial corticosteroid injection, rotator cuff and scapular stabilizer physiotherapy. Consider arthroscopic intervention if symptoms persist.
3. **Estimated Return-to-Sport Timeline:** Conservative: 6–8 weeks; Surgical: 4–6 months depending on procedure and rehabilitation.

**GPT-5:**

1. **Most likely diagnosis:** Partial-thickness bursal-side tear of the supraspinatus tendon with biceps tendon groove inflammation and AC joint degeneration.
2. **Recommended treatment plan:**
   - Conservative (first-line): Rest from aggravating activities, NSAIDs, physiotherapy (rotator cuff and scapular stabilizer strengthening, stretching), corticosteroid injection for persistent pain.
   - Surgical (if symptoms persist >3–6 months or functional limitation significant): Arthroscopic debridement or repair of the supraspinatus, possible biceps tenodesis, and AC joint decompression if indicated.
3. **Estimated return-to-sport timeline:**
   - Conservative: 6–12 weeks.
   - Surgical: 4–6 months, depending on repair integrity and functional recovery.

**Case 3**

**Age:** 15 years
**Gender:** Male
**Sport:** Basketball youth player
**History:** Pain in the left knee after training and matches.
**Physical Examination:** Tenderness and palpable firmness at the left tibial tuberosity.
**Radiology:** X-ray shows osteochondrosis in the tibial tuberosity.

**Literature-Based Recommendation – Osgood–Schlatter Disease (Adolescents)**
“Osgood–Schlatter disease is a self-limiting apophysitis commonly seen in adolescents. Management should be conservative, including activity modification based on pain level, ice, NSAIDs, and stretching/strengthening of quadriceps and hamstrings. Radiologic imaging is rarely needed, and surgical intervention is reserved only for persistent disabling symptoms.”
*Reference:* Smith JM. *StatPearls* [Internet]. 2023.

**GPT-4:**

- **Most Likely Diagnosis:** Osgood–Schlatter disease (tibial tuberosity apophysitis)
- **Recommended Treatment Plan:** Conservative—activity modification, ice, NSAIDs if needed, quadriceps and hamstring stretching.
- **Estimated Return-to-Sport Timeline:** 2–8 weeks depending on symptom resolution.

**GPT-5:**

1. **Most likely diagnosis:** Osgood–Schlatter disease.
2. **Recommended treatment plan:** Activity modification, ice, NSAIDs, stretching and strengthening of quadriceps and hamstrings.
3. **Estimated return-to-sport timeline:** Usually 4–8 weeks, depending on symptom resolution.

**Case 4**

**Age:** 13 years
**Gender:** Male
**Sport:** Football youth player
**History:** Pain in the left foot sole after training and matches.
**Physical Examination:** Tenderness at the posterior calcaneus of the left foot, no swelling.
**Radiology:** X-ray shows increased sclerosis and fragmentation in the calcaneal apophysis.

**ESSKA Recommendation – Calcaneal Apophysitis (Sever’s Disease)**
“In young athletes presenting with heel pain and radiographic signs of calcaneal apophysitis, conservative treatment is recommended. This includes relative rest, heel lifts or cushions to offload the apophysis, and physiotherapy focused on stretching of the posterior chain. No role for imaging-based follow-up or surgical treatment is indicated in typical cases. Sever’s disease is a self-limiting condition and should be managed conservatively. Emphasis should be placed on load management and muscle-tendon flexibility.”
*Reference:* Maffulli N, et al. *Knee Surg Sports Traumatol Arthrosc.* 2018;26(6):1884–1894. <https://doi.org/10.1007/s00167-017-4719-5>

**GPT-4:**

- **Most Likely Diagnosis:** Sever’s disease (calcaneal apophysitis)
- **Recommended Treatment Plan:** Conservative—activity modification, heel pads or orthotics, ice, NSAIDs, stretching of the Achilles tendon.
- **Estimated Return-to-Sport Timeline:** 2–6 weeks depending on symptom resolution; gradual return as tolerated.

**GPT-5:**

- **Diagnosis:** Sever’s disease (calcaneal apophysitis).
- **Treatment:** Activity modification, heel cups or heel pads, calf stretching, ice application, NSAIDs if needed.
- **Return-to-sport:** Typically 2–8 weeks, depending on symptom resolution.

**Case 5**

**Age:** 17 years
**Gender:** Female
**Sport:** Youth volleyball player
**History:** Twisting injury to the left kneecap 4 months ago, treated conservatively. Recurrence during training followed by a fall.
**Physical Examination:** Painful and swollen left knee, unclear exam due to pain.
**Radiology:** X-ray normal. MRI shows intense edema around the MPFL and patella, possible full-thickness tear.

**ESSKA Recommendation – MPFL Injury in Adolescents**
“In skeletally immature athletes with recurrent patellar dislocation and MRI evidence of MPFL injury, early surgical reconstruction is recommended in the presence of high-risk anatomical factors (e.g., trochlear dysplasia, patella alta, increased TT–TG distance). In first-time dislocations without high-risk features, conservative management with structured physiotherapy focusing on quadriceps and hip strengthening is advised. Return to sport is allowed only after restoration of quadriceps strength, dynamic stability, and full pain-free range of motion, generally not before 4–6 months post-surgery or 2–3 months with conservative treatment.”
*Reference:* Dejour D, Grimberg J, Neyret P, et al. *Knee Surg Sports Traumatol Arthrosc.* 2021;29(11):3290–3303. <https://doi.org/10.1007/s00167-021-06574-4>

**GPT-4:**

1. **Most Likely Diagnosis:** Recurrent patellar instability with suspected MPFL tear.
2. **Recommended Treatment Plan:** Start with conservative management (brace, physiotherapy focusing on quadriceps and core strengthening). Surgical MPFL reconstruction should be considered due to recurrence and MRI findings.
3. **Estimated Return-to-Sport Timeline:** Conservative: 6–8 weeks if stable and symptoms improve; Surgical: 4–6 months postoperatively with structured rehabilitation.

**GPT-5:**

- **Diagnosis:** Recurrent patellar dislocation with possible MPFL tear.
- **Treatment:** Initial immobilization, physiotherapy for quadriceps and hip strengthening; surgical MPFL reconstruction considered due to recurrence.
- **Return-to-sport:** Typically 4–6 months post-surgery, following phased rehabilitation.

**Case 6**

**Age:** 15 years
**Gender:** Male
**Sport:** Youth football player
**History:** Right knee pain and swelling after a twisting injury.
**Physical Examination:** Painful and restricted movement, suprapatellar swelling.
**Radiology:** MRI shows near-total ACL tear, longitudinal tear of medial meniscus, edema in the lateral femoral condyle.

**ESSKA Recommendation – ACL Tear with Concomitant Meniscus Injury in Adolescents**
“In skeletally immature or adolescent athletes with ACL rupture, early surgical stabilization is recommended to reduce the risk of secondary meniscal and chondral damage. Concomitant meniscus repair should be performed whenever possible to preserve tissue. In partial or near-total ACL injuries with high functional demands, surgical reconstruction is preferred over conservative care. Return to pivoting sports should follow a criterion-based rehabilitation protocol, typically no earlier than 9 months after surgery.”
*References:*

1. Kocher MS, et al. *Knee Surg Sports Traumatol Arthrosc.* 2020;28(8):2606–2620. doi:10.1007/s00167-020-06018-y
2. Arundale AJH, et al. *Knee Surg Sports Traumatol Arthrosc.* 2022;30(4):1033–1049. doi:10.1007/s00167-022-06919-9

**GPT-4:**

- **Most Likely Diagnosis:** Near-complete ACL tear with medial meniscus longitudinal tear and bone contusion (lateral femoral condyle).
- **Recommended Treatment Plan:** ACL reconstruction with meniscal repair is recommended due to functional instability and associated injuries. Surgery may be delayed depending on growth plate status.
- **Estimated Return-to-Sport Timeline:** Approximately 9–12 months post-surgery with progressive rehabilitation.

**GPT-5:**

1. **Most likely diagnosis:** Near-total ACL tear with longitudinal medial meniscus tear and bone bruise in the lateral femoral condyle.
2. **Recommended treatment plan:** Surgical ACL reconstruction with meniscus repair if feasible; prehabilitation before surgery; postoperative structured rehabilitation program.
3. **Estimated return-to-sport timeline:** ~9–12 months.

**Case 7**

**Age:** 33 years
**Gender:** Male
**Sport:** Professional football player
**History:** Previous partial meniscectomy on the right knee, intermittent pain.
**Physical Examination:** Normal movements, medial McMurray test (+), no swelling, no crepitus.
**Radiology:** X-ray shows 20° varus deformity, KL grade 2 medial OA. MRI shows degenerative medial meniscus tear, osteochondral defect.

**ESSKA Recommendation – Degenerative Medial Meniscus Tear with Varus Alignment and Early OA**
“In patients with degenerative medial meniscus tears associated with varus malalignment and early medial compartment osteoarthritis, meniscus preservation should be prioritized when possible. For symptomatic cases after partial meniscectomy, high tibial osteotomy (HTO) is recommended to correct alignment, reduce load on the medial compartment, and potentially slow OA progression. HTO may be combined with cartilage or meniscus restorative procedures when indicated.”
*Reference:* Beaufils P, et al. *Knee Surg Sports Traumatol Arthrosc.* 2017;25(2):335–346. <https://doi.org/10.1007/s00167-016-4400-3>

**GPT-4:**

1. **Most Likely Diagnosis:** Degenerative medial meniscus tear with medial compartment osteoarthritis (KL grade 2) and osteochondral defect, with 20° varus malalignment.
2. **Recommended Treatment Plan:** Initial conservative approach (physiotherapy, NSAIDs, unloading brace, activity modification). If symptoms persist, consider HTO for alignment correction and joint preservation.
3. **Estimated Return-to-Sport Timeline:** Conservative: ~6–8 weeks; Post-HTO: ~6–9 months.

**GPT-5:**

- **Diagnosis:** Degenerative medial meniscus tear with osteochondral defect and medial compartment OA (KL grade 2) in a varus-aligned knee.
- **Treatment:** Conservative management initially; consider HTO with/without cartilage restoration if symptoms persist.
- **Return-to-sport:** Variable; conservative 2–3 months; surgical correction 6–12 months.

**Case 8**

**Age:** 24 years
**Gender:** Male
**Sport:** Ice hockey player
**History:** Previous clavicle fracture surgery, sudden pain in right thigh during match.
**Physical Examination:** Swelling and tenderness in anteromedial thigh.
**Radiology:** MRI shows extensive hematoma in medial thigh muscles, pubic bone edema.

**ESSKA Recommendation – Adductor Muscle Injury in Athletes**
“In cases of adductor muscle strain with hematoma formation, conservative treatment is recommended as first-line management (RICE), followed by progressive physiotherapy focusing on hip adductors and core stability. Surgical intervention is rarely needed.”
*Reference:* Weir A, Brukner P, Delahunt E, et al. *Knee Surg Sports Traumatol Arthrosc.* 2015;23(10):334–345. <https://doi.org/10.1007/s00167-015-3541-y>

**GPT-4:**

1. **Most Likely Diagnosis:** Adductor muscle strain or partial tear with intramuscular hematoma and pubic bone stress reaction (adductor-related groin pain).
2. **Recommended Treatment Plan:** Conservative—rest, ice, NSAIDs, compression; gradual physiotherapy (core and adductor strengthening); monitor for chronic groin pain/sports hernia if symptoms persist.
3. **Estimated Return-to-Sport Timeline:** Mild/moderate 4–6 weeks; severe/recurrent 6–10 weeks.

**GPT-5:**

- **Diagnosis:** Adductor muscle strain with intramuscular hematoma and pubic bone stress reaction.
- **Treatment:** RICE; gradual physiotherapy; avoid aggravating activities.
- **Return-to-sport:** ~4–6 weeks.

**Case 9**

**Age:** 28 years
**Gender:** Male
**Sport:** Professional football player
**History:** Occasional pain in left hip.
**Physical Examination:** Pain in hip flexion; otherwise full ROM.
**Radiology:** MRI shows degenerative anterior labral tear.

**ESSKA Recommendation – FAI with Labral Tear in Athletes**
“In symptomatic athletes with femoroacetabular impingement (FAI) and labral tears unresponsive to conservative treatment, arthroscopic correction of the bony deformity and labral repair is recommended.”
*Reference:* Grammatopoulos G, et al. *Knee Surg Sports Traumatol Arthrosc.* 2021;29(1):11–23.

**GPT-4:**

1. **Most Likely Diagnosis:** Degenerative anterior labral tear of the left hip.
2. **Recommended Treatment Plan:** Start with conservative management (activity modification, NSAIDs, physiotherapy focusing on hip stability and core strengthening). If symptoms persist, consider hip arthroscopy for labral debridement or repair.
3. **Estimated Return-to-Sport Timeline:** Conservative ~4–6 weeks; Surgical ~3–6 months.

**GPT-5:**

- **Diagnosis:** Degenerative anterior acetabular labral tear (left hip).
- **Treatment:** Initial conservative care; arthroscopic labral repair/debridement if symptoms persist.
- **Return-to-sport:** ~8–12 weeks after surgical repair; shorter (4–6 weeks) if conservative and symptoms resolve.

**Case 10**

**Age:** 28 years
**Gender:** Female
**Sport:** Professional boxer
**History:** Sudden right shoulder pain during training; dislocation reduced on-site.
**Physical Examination:** Inability to move shoulder due to pain.
**Radiology:** MRI shows anterior inferior labral tear (Bankart lesion).

**ESSKA Recommendation – Traumatic Anterior Shoulder Instability with Bankart Lesion in Athletes**
“In young athletes with a first-time traumatic anterior shoulder dislocation and a confirmed Bankart lesion, early arthroscopic Bankart repair is recommended to reduce recurrence risk.”
*Reference:* Olds M, et al. *Knee Surg Sports Traumatol Arthrosc.* 2020;28(7):2031–2048. <http://doi.org/10.1007/s00167-020-06020-6>

**GPT-4:**

1. **Most Likely Diagnosis:** Traumatic anterior shoulder dislocation with Bankart lesion.
2. **Recommended Treatment Plan:** Initial sling (1–2 weeks), NSAIDs, physiotherapy for ROM/strength; definitive arthroscopic Bankart repair for high-demand athletes.
3. **Estimated Return-to-Sport Timeline:** Surgical 4–6 months; Non-surgical 8–12 weeks (higher recurrence risk).

**GPT-5:**

1. **Most likely diagnosis:** Traumatic anterior shoulder dislocation with Bankart lesion.
2. **Recommended treatment plan:** Initial sling 1–2 weeks, NSAIDs, gradual physiotherapy; arthroscopic Bankart repair recommended for high-demand athletes.
3. **Estimated return-to-sport timeline:** Post-surgical ~4–6 months; Non-surgical 8–12 weeks (higher recurrence risk).

**Case 11**

**Age:** 22 years
**Gender:** Male
**Sport:** Marathon runner
**History:** Sudden swelling and pain in right leg after training.
**Radiology:** MRI shows bone edema in midshaft tibia, increased signal in lateral muscle groups.

**Literature Recommendation – Tibial Stress Injuries in Endurance Athletes**
“Initial treatment should be conservative (rest, load modification, physiotherapy). MRI is preferred for early diagnosis and grading. Surgery is not indicated for bone stress injuries; fasciotomy only if CECS persists despite comprehensive conservative treatment.”
*References:* Bennell KL, et al. *KSSTA.* 2018;26(3):793–802. doi:10.1007/s00167-018-4868-6 • Schepsis AA, et al. *Curr Rev Musculoskelet Med.* 2022;15(4):375–384.

**GPT-4:**

1. **Most Likely Diagnosis:** Midshaft tibial stress reaction with associated lateral compartment strain/edema.
2. **Recommended Treatment Plan:** Stop running; relative rest, ice, NSAIDs; gradual weight-bearing; physiotherapy; address risk factors.
3. **Estimated Return-to-Sport Timeline:** ~6–8 weeks (mild/moderate); up to 12 weeks if severe.

**GPT-5:**

- **Most likely diagnosis:** Tibial stress fracture with associated muscular strain/edema.
- **Recommended treatment plan:** Activity modification and rest; protective weight-bearing if needed; physiotherapy; address contributing factors.
- **Estimated return-to-sport timeline:** Mild/moderate 6–8 weeks; severe up to 12 weeks.

**Case 12**

**Age:** 16 years
**Gender:** Male
**Sport:** Professional football player
**History:** Right knee pain for 1 month, worsening in last week.
**Physical Examination:** Pain in flexion.
**Radiology:** X-ray shows fissure-like lesion in medial femoral condyle. MRI shows stage 2 osteochondritis dissecans.

**ESSKA Recommendation – Juvenile Osteochondritis Dissecans (Stage 2)**
“Stable stage 1–2 OCD in skeletally immature athletes: first-line non-operative (activity modification, partial weight-bearing, physiotherapy). Radiological monitoring every 3–4 months. Consider surgery if no improvement after 6 months or if instability develops.”
*Reference:* Kocher MS, et al. *Knee Surg Sports Traumatol Arthrosc.* 2020;28(2):984–994. <https://doi.org/10.1007/s00167-019-05643-1>

**GPT-4:**

- **Most Likely Diagnosis:** Stage 2 OCD of the medial femoral condyle.
- **Recommended Treatment Plan:** Activity restriction; NSAIDs; physiotherapy (strengthening, proprioception); serial MRI; consider arthroscopic drilling/fixation if persistent.
- **Estimated Return-to-Sport Timeline:** Conservative 3–6 months; Surgical 6–9 months.

**GPT-5:**

1. **Most likely diagnosis:** Stage 2 OCD (medial femoral condyle, right knee).
2. **Recommended treatment plan:** Activity restriction; NSAIDs; quadriceps strengthening; proprioception; periodic MRI follow-up; consider arthroscopy if persistent.
3. **Estimated return-to-sport timeline:** Conservative 3–6 months; Surgical 6–9 months.

**Case 13**

**Age:** 21 years
**Gender:** Male
**Sport:** Professional handball player
**History:** Pain and difficulty bearing weight on right ankle.
**Physical Examination:** Swelling in anterior dorsal and lateral ankle, painful movements.
**Radiology:** MRI shows complete ATFL and CFL tears.

**Literature Recommendation – Acute Lateral Ankle Ligament Injuries (Complete ATFL + CFL) in Athletes**
“First-line: early functional management (brace/taping, protected weight-bearing, early ROM, neuromuscular training). Surgical repair may be considered in elite/high-demand athletes or persistent mechanical instability.”
*References:* van den Bekerom MPJ, et al. *KSSTA.* 2013;21(6):1390–1395 • White WJ, et al. *KSSTA.* 2016 • Hong CC, Calder JD. *KSSTA.* 2023.

**GPT-4:**

1. **Most Likely Diagnosis:** Acute grade III lateral ankle sprain (complete ATFL and CFL tear).
2. **Recommended Treatment Plan:** Primary functional rehabilitation; surgery for elite athletes or persistent instability.
3. **Estimated Return-to-Sport Timeline:** Conservative 8–10 weeks; Surgical 3–4 months.

**GPT-5:**

- **Diagnosis:** Acute grade III lateral ankle ligament injury (complete ATFL + CFL).
- **Treatment:** Functional rehabilitation; surgery only for elite/failed rehab.
- **Return-to-sport:** Conservative 8–10 weeks; Surgical 3–4 months.

**Case 14**

**Age:** 25 years
**Gender:** Male
**Sport:** Professional football player
**History:** ACL reconstruction 2 years ago with ipsilateral hamstring graft; recurrent instability and pain after twisting injury.
**Physical Examination:** Swelling, painful movements, anterior drawer (+).
**Radiology:** MRI shows ACL graft rupture.

**ESSKA Recommendation – ACL Graft Failure in Elite Athletes**
“In athletes with symptomatic ACL graft rupture and objective instability, revision ACL reconstruction is recommended; address tunnel malposition, concomitant injury, or malalignment. Graft choice individualized; autograft often preferred.”
*Reference:* Grassi A, et al. *Knee Surg Sports Traumatol Arthrosc.* 2022;30(2):452–470. <https://doi.org/10.1007/s00167-021-06738-x>

**GPT-4:**

1. **Most Likely Diagnosis:** ACL graft rupture (failed ACL reconstruction).
2. **Recommended Treatment Plan:** Revision ACL reconstruction (autograft/allograft), structured rehabilitation.
3. **Estimated Return-to-Sport Timeline:** ~9–12 months.

**GPT-5:**

- **Diagnosis:** ACL graft rupture (failed ACL reconstruction).
- **Treatment:** Revision ACL reconstruction; concurrent rehab.
- **Return-to-sport:** ~9–12 months.

**Case 15**

**Age:** 23 years
**Gender:** Male
**Sport:** Baseball pitcher (right-handed)
**History:** Intermittent right shoulder pain.
**Physical Examination:** Normal ROM, localized pain over biceps tendon.
**Radiology:** MRI shows stage 2 SLAP tear, anterior inferior labral degeneration, biceps tendon edema.

**ESSKA Recommendation – SLAP Tear and Biceps Tendon Pathology in Overhead Athletes**
“In symptomatic type II SLAP lesions, arthroscopic SLAP repair or biceps tenodesis should be considered depending on age, activity level, and associated pathology.”
*Reference:* Andrews JR, Carson WG, Ortega K, et al. *Knee Surg Sports Traumatol Arthrosc.* 2018;26(1):196–206. <https://doi.org/10.1007/s00167-017-4744-4>

**GPT-4:**

1. **Most Likely Diagnosis:** Stage 2 SLAP tear with anterior inferior labral degeneration and biceps tendinopathy.
2. **Recommended Treatment Plan:** Start with conservative management (rest, NSAIDs, physiotherapy, throwing mechanics correction). If persistent after 3–4 months, consider arthroscopic SLAP repair or biceps tenodesis.
3. **Estimated Return-to-Sport Timeline:** Conservative ~3–4 months; Surgical ~6–9 months.

**GPT-5:**

1. **Most Likely Diagnosis:** Stage 2 SLAP tear with anterior inferior labral degeneration and biceps tendinopathy.
2. **Recommended Treatment Plan:** Initial conservative management; if persistent, consider arthroscopic SLAP repair or biceps tenodesis.
3. **Estimated Return-to-Sport Timeline:** Conservative 3–4 months; Surgical 6–9 months.
